# Pharmacokinetics and Quantitative Structure–Pharmacokinetics Relationship Study of Xanthine Derivatives with Antidepressant, Anti-Inflammatory, and Analgesic Activity in Rats

**DOI:** 10.3390/pharmaceutics16111463

**Published:** 2024-11-16

**Authors:** Artur Świerczek, Małgorzata Szafarz, Agnieszka Cios, Jan Kobierski, Krzysztof Pociecha, Daniel Attard Saliba, Grażyna Chłoń-Rzepa, Elżbieta Wyska

**Affiliations:** 1 Department of Pharmacokinetics and Physical Pharmacy, Faculty of Pharmacy, Jagiellonian University Medical College, 9 Medyczna Street, 30-688 Kraków, Poland; artur.swierczek@uj.edu.pl (A.Ś.);; 2 Department of Pharmacy, Faculty of Medicine and Surgery, University of Malta, MSD 2080 Msida, Malta; 3 Department of Medicinal Chemistry, Faculty of Pharmacy, Jagiellonian University Medical College, 9 Medyczna Street, 30-688 Kraków, Poland; grazyna.chlon-rzepa@uj.edu.pl

**Keywords:** quantitative structure–pharmacokinetics relationship, multivariate data analysis, regression analysis, methylxanthines, 7- and 7,8-substituted theophylline derivatives

## Abstract

Objective: The aim of this study was to develop quantitative structure–pharmacokinetics relationship (QSPKR) models for a group of xanthine derivatives with proven pharmacological activity and to investigate its applicability for the prediction of the pharmacokinetics of these compounds. Methods: The SYBYL-X, KowWin, and MarvinSketch programs were employed to generate a total of fourteen descriptor variables for a series of new compounds: 7- and 7,8-substituted theophylline derivatives (GR-1–GR-8) and three well-known methylxanthines. Pharmacokinetic profiles of all compounds were determined after intravenous administration of studied compounds to cannulated male rats. Pharmacokinetic parameters were calculated using noncompartmental analysis. Results: Multiple linear regression revealed that log*D* was the main determinant of the variability in *V_ss_*, *λ_z_*, and *CL* of the studied compounds. Moreover, *λ_z_* and *CL* depended on LUMO and HEFO, while for *V_z_* COAR was the only explanatory variable. The developed QSPKR models accounted for most of the variation in *V_ss_*, *λ_z_*, *CL*, and fraction unbound (*f_u_*) (*R*^2^ ranged from 0.68 to 0.91). Cross-validation confirmed the predictive ability of the models (*Q*^2^ = 0.60, 0.71, 0.34, and 0.32 for *V_ss_*, *λ_z_*, *CL*, and f_u_, respectively). Conclusions: The multivariate QSPKR models developed in this study adequately predicted the overall pharmacokinetic behavior of xanthine derivatives in rats.

## 1. Introduction

Advancements in computer science made it possible to evolve from qualitative to quantitative structure–activity relationships (QSAR). This approach is mainly used by medicinal chemists in drug design, and in most cases, it is based on in vitro data. However, it is well known that relationships derived from compounds investigated in vitro may not apply to in vivo situations. One of the key factors responsible for the lack of correlations between in vitro and in vivo data is related to the pharmacokinetics of studied compounds [1].

It is well known that pharmacokinetic parameters are strongly correlated with the physicochemical properties of a drug. One of the most important parameters is the logarithm of the octanol/water partition coefficient (log*P* or log*Kow*) or the distribution coefficient (log*D*). The literature data indicate that both plasma protein binding and volume of distribution are dependent on the lipophilicity of a compound [2]. The implementation of pharmacokinetic parameters in QSAR studies is a step towards a more rational drug design. It may facilitate the design of compounds that will be characterized by optimal pharmacologic effect and low toxicity, accelerating the identification of new drug candidates and reducing the cost of the drug development process [1,3].

This type of approach is very important for the pharmaceutical industry, as the implementation of combinatorial chemistry has increased the number of compounds entering the drug discovery process. However, the large number of drug candidates forces an early selection of the compounds that have the greatest possibility of success. In this aspect, the focus is not only on achieving the best pharmacological efficacy but also on seeking desirable ADME (absorption, distribution, metabolism, excretion) characteristics [4,5,6]. A variety of high-throughput experimental and theoretical methods have arisen for screening candidate molecules, among which the development of quantitative structure–pharmacokinetics relationships (QSPKR) using ‘in silico’ procedures is of special interest. QSPKR models focus on the association of structural features of compounds with pharmacokinetic parameters [7]. These relationships allow for the prediction of the pharmacokinetic behavior of new compounds from their easily measured or estimated physicochemical or molecular properties.

Xanthines are purine analogs that are inhibitors of phosphodiesterases and antagonists of adenosine receptors. Up to date, different modifications of the xanthine scaffold led to obtaining compounds with a wide range of activities. For example, istradefylline has been used for the treatment of Parkinson’s disease, and pentoxifylline (PTX) has been effective in the management of peripheral vascular disease and off-label in newborn infants with sepsis. Lisofylline (a metabolite of PTX) has been tested for use in acute lung damage, acute respiratory distress syndrome, and diabetes mellitus. In turn, propentofylline has proved effective in clinical trials in patients with vascular dementia, whereas theophylline (Theo) is widely used to treat asthma and chronic obstructive pulmonary disease [8]. With its versatile and structurally rigid scaffold, xanthine provides an excellent starting point for the synthesis of more effective derivatives affecting different molecular targets [9]. Despite the fact that a relatively large number of animal and human studies of this group of compounds have been described in the literature, much less is known about their pharmacokinetics. Therefore, xanthine derivatives were selected in this study to build QSPKR models to assess which of the molecular descriptors correlate best with the pharmacokinetic parameters of these compounds and to predict the pharmacokinetic properties of new xanthine derivatives using their structural features.

**Figure 1:**
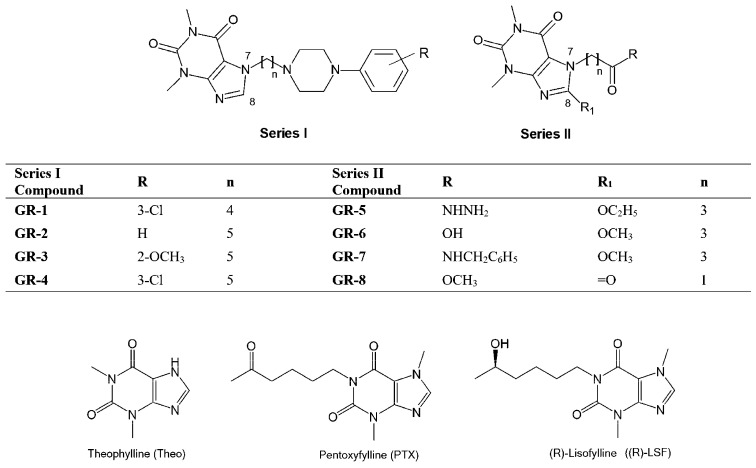
Chemical structures of evaluated methylxanthines: 7-substituted (GR-1–GR-4, series I) and 7,8-disubstituted (GR-5–GR-8, series II) theophylline derivatives, theophylline (Theo), pentoxifylline (PTX), and (R)-lisofylline ((R)-LSF).

The first aim of this study was to evaluate the pharmacokinetics of previously reported two series of pharmacologically active xanthine derivatives following intravenous administration in rats. Series I comprised 7-phenylpiperazinylalkyl theophylline derivatives, the potent 5-HT_1A_ ligands with diversified (pre- and/or post-synaptic agonists or postsynaptic antagonism) functional profiles and antidepressant and/or anxiolytic-like properties (GR-1–GR-4; Figure 1) [10]. Series II presented theophyllin-7-yl derivatives of butanoic (GR-5–GR-7; Figure 1) and acetic (GR-8; Figure 1) acids with additional alkoxy or oxo substituent in position 8 with analgesic and anti-inflammatory activities [11,12,13]. In addition, the pharmacokinetic parameters of Theo and two other well-known methylxanthines: PTX and R-lisofylline ((R)-LSF) (Figure 1) with proven anti-inflammatory properties were estimated. The second aim was to study the quantitative relationship between the structure and pharmacokinetic parameters of Theo, its derivatives (GR-1 to GR-8), and other methylxanthines (PTX, (R)-LSF) using the forward stepwise inclusion method coupled with multiple linear regression.

## 2. Materials and Methods

### 2.1. Chemicals and Reagents

Most studied compounds were synthesized in the Department of Medicinal Chemistry, Jagiellonian University Medical College, Cracow, Poland. The chemical structures are presented in Figure 1, and the synthesis is described in earlier publications [5,6]. Theo, theobromine (internal standard, IS), and PTX were from Sigma Aldrich (Darmstadt, Germany). (R)-LSF was obtained in the Department of Technology and Biotechnology of Drugs, Jagiellonian University Collegium Medicum. Temazepam (IS) was a gift from Polfa (Warszawa, Poland). Acetonitrile, hexane, ethyl acetate, and methanol of HPLC grade were also from Sigma Aldrich (Germany). Potassium dihydrogen phosphate and sulfuric acid (96%) were from Merck (Darmstadt, Germany). Sodium hydroxide (volumetric concentrate for dilution to 1 L) was obtained from J. T. Baker (Deventer, The Netherlands). Ultrapure deionized water (0.1 µS/cm) was prepared in-house using a Hydrolab water purification system (Straszyn, Poland) with microfiltration.

### 2.2. Pharmacokinetic Experiments

Male Wistar rats, 15–17 weeks of age and weighing between 250 and 350 g, purchased from the Animal Facility of the Jagiellonian University Medical College, Krakow, Poland, were used in all experiments. They were kept under conditions of constant temperature (21–25 °C) and relative humidity of approximately 40–65% with a standard light/dark cycle. Animals were housed in stainless steel cages with suspended wire mesh floors (maximum of 5 rats per cage) with free access to distilled water and the commercial rodent chaw. All study protocols were approved by the First Local Ethical Committee on Animal Testing of the Jagiellonian University (licenses no. Zi/604/2011 and 13/2014).

Three days prior to the experiment, the rat’s jugular vein was cannulated (SAI Infusion Technologies, Lake Villa, IL, USA), allowing for multiple blood samples from a single animal. Tested compounds were administered into tail vein as a single dose of 50 mg/kg Theo, 40 mg/kg PTX and LSF, and 5 mg/kg GR-1, GR-2, GR-3, GR-4, GR-5, and 2 mg/kg GR-6, GR-7, and GR-8 in 5% DMSO in sterile saline (Polpharma, Starogard Gdański, Poland). The doses were selected based on the results of our previous studies on rats and mice. Four rats per compound were used. Multiple blood samples were taken over a period of at least 120 min into heparinized tubes; plasma was separated by centrifugation (Universal 32, Hettich, Germany) at 1400× *g* for 20 min and stored at −80 °C until analysis.

The free concentrations of studied compounds in plasma were determined and unbound fraction (*f_u_*) was calculated in all available plasma samples obtained 15 min after the intravenous administration. At this time point, 500–600 µL of blood was collected and plasma samples obtained were divided into two portions: 150–200 µL for measurement of free compound concentrations and 100 µL for measurement of the total drug concentrations. Plasma proteins were separated by an ultrafiltration method using Centrifree^®^ devices with Ultracel PL regenerated cellulose membrane, 30,000 NMWL (Merck, Germany). For direct measurement of the free concentrations of compounds, each plasma sample was transferred to a sample reservoir of the disposable filter unit and centrifuged (1000× *g*) for 20 min at 25 °C. An aliquot of the ultrafiltrate was directly subjected to analysis. Free and total compound concentrations were measured by a high-performance liquid chromatography method with diode-array detection (HPLC-DAD) or liquid chromatography with tandem mass spectrometry (LC-MS/MS).

### 2.3. Quantification of Studied Compounds

For the purpose of the quantitative analysis of free and total plasma concentrations of studied compounds, different analytical methods were developed. Theo was extracted from the biological samples by liquid–liquid extraction using dichloromethane. The mixture consisting of 100 μL of rat plasma or ultrafiltrate, 10 μL of theobromine (25 μg/mL in methanol) used as IS, 20 μL of 0.1 M HCl, and 5 mL of dichloromethane was agitated on a shaker (VXR Vibrax, IKA, Staufen, Germany) and subsequently centrifuged for 20 min at 3000 rpm (Universal 32, Hettich, Germany). A clear supernatant (4 mL) was transferred into a glass conical tube and evaporated to dryness under a stream of nitrogen in a water bath at 37 °C. The residue was then reconstituted in 100 μL of methanol, vortexed for 30 s, and the solution was transferred into the automatic sampler vials. Finally, 10–50 μL aliquot was injected into the HPLC/DAD system (LaChrome Elite, Merck-Hitachi, Germany). Chromatographic separation was performed on the LiChrospher 100 RP-18 (250 × 4 mm i.d., 5 µm particle size) analytical column coupled with a LiChroCART^®^ guard column (4.0 × 4.0 mm, 5 μm) from Merck (Darmstadt, Germany). The mobile phase consisted of a mixture of methanol and deionized water (20:80, *v*/*v*) and was pumped through the system at a flow rate of 1.0 mL/min. The peaks were detected by a DAD detector at the wavelength of 271 nm. The average retention time was 18.2 min for Theo and 10.5 min for IS. The analytical run lasted 20 min.

Plasma or ultrafiltrate samples containing PTX or (R)-LSF were placed in a glass tube and spiked with 10 µL of the IS solution (100 µg/mL temazepam in methanol). Samples were then acidified with 20 µL of 0.1 M hydrochloric acid, and 3 mL of dichloromethane was added to each sample. Tubes were shaken for 20 min and subsequently centrifuged (2000× *g*, 20 min). Then, 2.5 mL of dichloromethane was transferred to a clean glass conical tube and evaporated under a gentle stream of nitrogen in a water bath at 37 °C. The dried residue was dissolved in 100 µL of the mobile phase, and 50 µL of this solution was injected into the HPLC column. Analysis was performed on a 250 × 4.6 mm Chiralpak AD column (Daicel Corp., Tokyo, Japan) with 10 μm particle size, protected with a 20 × 4.6 mm LC-Si guard-column (Supelco Inc., Bellefonte, PA, USA). Mobile phase consisted of hexane: 2-propanol, 84:16 (*v*/*v*), and 0.01% diethylamine and was pumped at a flow rate of 1.4 mL/min. Calibration curves were linear in a range of 0.1–50 mg/L. The retention times of IS, PTX, and (R)-LSF were 7, 16, and 20 min, respectively. HPLC system (Thermo Separation Product, San Jose, CA, USA) consisted of a UV/VIS spectrophotometric detector (Spectra 100) with absorbance set at 275 nm, P100 pump, and Rheodyne 7125 manual injector (Alltech Associates, Deerfield, IL, USA) with a 50 µL sample loop. Chromatograms were recorded and integrated with an SP 4400 ChromJet integrator (San Jose, CA, USA). Compounds GR-1, GR-2, GR-3, GR-4, GR-5, GR-6, GR-7, and GR-8 were quantified in plasma and ultrafiltrate using the LC-MS/MS system. Plasma samples were precipitated with 2 volumes of acetonitrile with the addition of 0.1% formic acid, briefly vortexed, and centrifuged at 10,000× *g* for 10 min (Eppendorf miniSpin centrifuge, Hamburg, Germany). The supernatants (10 μL) were injected into an HPLC system (Agilent 1100, Agilent Technologies, Waldbronn, Germany). Chromatography was performed on Waters XTerraTM 5 µm C18 50 × 3 mm analytical column with gradient elution using a mobile phase containing acetonitrile and water with 0.1% formic acid. The chromatographic run lasted 10 min, and detection was performed by an Applied Biosystems MDS Sciex API 2000 triple quadrupole mass spectrometer (Foster City, CA, USA) set at unit resolution. The mass spectrometer was operated in the selected reactions monitoring mode (SRM), monitoring the transition of the protonated molecular ions to their specific fragments. The ion source temperature was maintained at 500 °C. The ionspray voltage was set at 5000 V. The curtain gas (CUR) was set at 20 and the collision gas (CAD) at 5. The compound-dependent mass spectrometric conditions were optimized by continuously infusing the standard solutions at the rate of 5 μL/min using a Harvard infusion pump. Data acquisition and processing were accomplished using the Applied Biosystems Analyst version 1.4.2 software. Calibration curves were linear from 1 to 2000 ng/mL for compounds GR-1, GR-2, and GR-5 to GR-7 and from 10 to 2000 ng/mL for compounds GR-3, GR-4, and GR-8.

All developed analytical methods were validated according to procedures and acceptance criteria recommended for bioanalytical method validation for pharmacokinetic studies regarding selectivity and specificity, linearity, intra- and inter-day accuracy and precision, limit of detection and quantitation, as well as recovery and stability [14]. The highest intra- and interday accuracy calculated as RSD% was observed for compound GR-8. The values of these parameters ranged from 95.06 to 100.96% and from 94.89 to 106.95%, respectively. In turn, the lowest accuracy, though still within recommended ranges, was obtained for GR-5, with values ranging from 89.65 to 113.54% in single day and from 88.75 to 108.28% across different days. The highest precision was observed for compound GR-8 (from 0.14 to 5.81%) and the lowest for GR-1 (from 0.91 to 9.42%). Stability of the investigated compounds (short-term, benchtop, and autosampler) under above-mentioned conditions was satisfactory since the deviation from nominal value was within ±15.0%.

### 2.4. Molecular Modeling

All the structures were constructed using the 2D sketcher module in SYBYL-X molecular modeling software (Tripos, Inc., version 1.2, St. Louis, MO, USA). The energy of minimization was carried out using the Powell method in MAXIMIN2 force field minimizer to gradient convergence, followed by MOPAC Austin Model 1 (AM1) optimization.

Molecular descriptors were generated by SYBYL-X in MOPAC module using the AM1 singlet method with time limit of 1 h for convergence. The electronic descriptors calculated in this study were the EE, NOFL, TOEN, HEFO, LUMO, HOMO, DIPO, and IOPO. The steric parameters were MW, CCR, COAR, and COVO.

One hydrophobic descriptor, the n-octanol-water partition coefficient (log*Kow*), was calculated using the KowWin program (Syracuse Research Corporation, Syracuse, NY, USA), whereas the distribution coefficient (log*D*) was calculated according to the equation:

(1) $$logD=logKow+log\frac{1}{1+10^{7.4-pKa}}$$

where ionization constant (*pKa*) values were obtained using MarvinSketch v. 15.10 (ChemAxon Ltd., Budapest, Hungary).

### 2.5. Data Analysis

Pharmacokinetic parameters were calculated based on the plasma concentration vs. time profiles of the tested compounds using noncompartmental analysis with the aid of Phoenix WinNonlin v. 6.3 (Pharsight Corp., Mountain View, CA, USA) software.

In order to find models describing quantitative structure—pharmacokinetic parameter relationships (QSPKR), an automatic forward stepwise inclusion method together with a multiple linear regression was used. Forward stepwise inclusion method assumes successively adding to the list of independent variables included in the model those variables (descriptors) that have the greatest impact on the dependent variable (pharmacokinetic parameter). To verify hypotheses for candidate variables, the F Snedecor statistics were used. The F-to-enter value, indicating how significant the contribution of the variable must be that it can be introduced into the model, was taken at the level of 3.8 (which approximately corresponds to α = 0.05). The F-to-remove value, specifying how low the significance for regression must be that the variable can be removed from the model, was established at the level of 2.7 (which approximately corresponds to α = 0.10) [15]. The predictability of the final models was tested by cross-validation using the “leave-one-out” method (LOOCV). In this procedure, all descriptors of one drug are omitted from the dataset. Then the model is rederived and used to predict pharmacokinetic parameters (y). Procedure is repeated until all drugs are omitted once [16,17].

In the present work, partial least squares (PLS) was used to develop QSPKR models for xanthine derivatives. This method is a kind of generalization of a common multiple linear regression and can be understood as a least squares regression extension of principal component analysis [16]. Partial least squares model can be expressed by matrix of regression coefficients $b_{PLS}$:

(2) $$Y=Xb_{PLS}+F,$$

where ***Y*** is a vector of dependent variables (pharmacokinetic parameters), ***X*** is a vector of independent variables (descriptors), and ***F*** is a residual vector [16].

Predictivity of the obtained models was quantified with the cross-validated $R^{2}$, also called $Q^{2}$, calculated according to the following expression:

(3) $$Q^{2}=1-\frac{\sum {\left(y_{pred}-y_{obs}\right)}^{2}}{\sum {\left(y_{obs}-y_{mean}\right)}^{2}}$$

A $Q^{2}$ value close to 1 indicates good predictive performance of the model, whereas negative $Q^{2}$ value suggests that model does not predict better than chance alone [17]. It is assumed that high $Q^{2}$ (i.e., $Q^{2}$ > 0.5) is a proof of high predictivity [18].

Multiple linear regression was performed using STATISTICA 12 (StatSoft, Tulsa, OK, USA), whereas partial least squares regression was conducted using STATGRAPHICS Centurion XVII (Statpoint Technologies, Warrenton, VA, USA). To find mathematical equation describing the unbound volume of distribution and lipophilicity relationship, exponential growth was fitted using OriginPro 9.1 (OriginLab, Northampton, MA, USA).

## 3. Results

Pharmacokinetic parameters of studied methylxanthines, i.e., Theo, its derivatives (GR-1 to GR-8) as well as PTX and (R)-LSF estimated using non-compartmental analysis are shown in Table 1.

All molecular descriptors calculated using SYBYL-X and KowWin programs are listed in Table 2. To obtain Log*Kow* values, the atom/fragment contribution method was used and adopted in the KowWin program. For the physicochemical descriptors obtained from the molecular modeling, a Pearson correlation matrix was calculated. A linear correlation was found for COAR and COVO, MW, CCR, NOFL, and EE parameters (R > 0.95, *p* < 0.05); therefore only COAR was included in the analysis. A forward stepwise inclusion method for multiple linear regression with HOMO, LUMO, HEFO, IOPO, DIPO, TOEN, COAR, log*Kow*, and log*D* as independent variables was used. This analysis gave models of *V_z_*, *V_ss_*, *λ_z_*, and *CL*. A summary of the equations describing QSPKR models with corresponding *R*^2^ and *Q*^2^ is shown in Table 3.

These models reveal significant correlations (*R*^2^ range from 0.72 to 0.91) as well as a good predictive performance for *V_z_*, *V_ss_*, and *λ_z_* (*Q*^2^ from 0.60 to 0.71) and an average predictive performance (*Q*^2^ = 0.34) for *CL*. The log*D* parameter appeared as the main determinant of the variability in *V_ss_*, *λ_z_*, and *CL* values. Moreover, *λ_z_* and *CL* depended on LUMO and HEFO values. In the case of *V_z_*, COAR is the only explanatory variable (Table 3).

**Figure 2:**
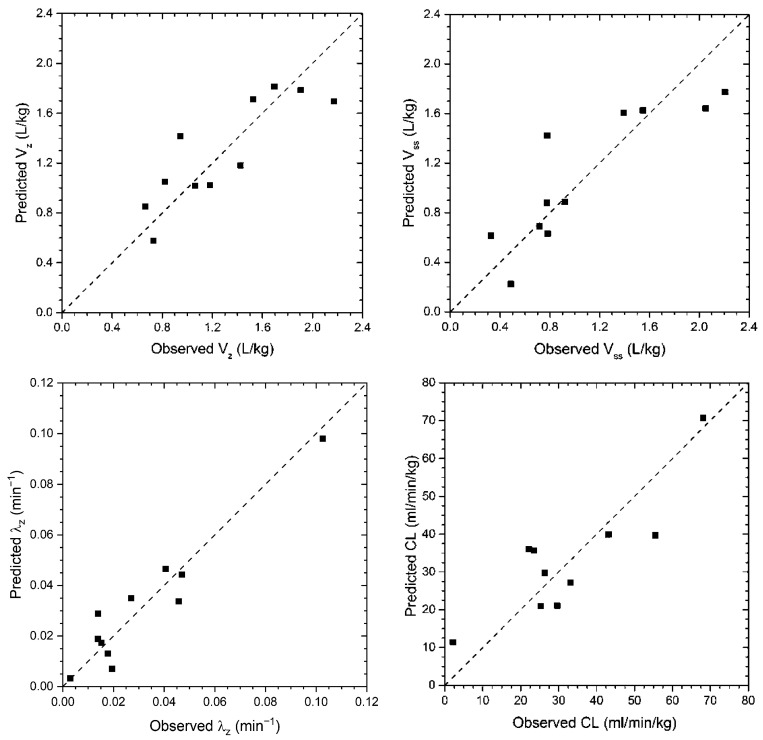
Predicted vs. actual (observed) *V_z_*, *V_ss_*, *λ_z_*, and *CL* values (black squares) with the lines of identity.

The predicted pharmacokinetic parameters demonstrated a high agreement with the observed parameters, as can be seen in Figure 2.

**Figure 3:**
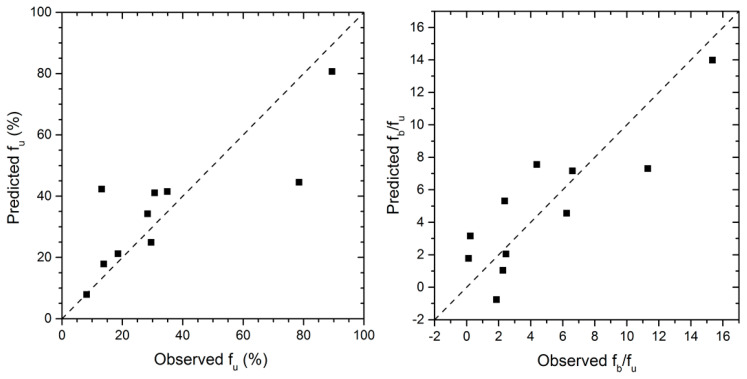
Predicted vs. actual (observed) *f_u_* and *f_b_*/*f_u_* values (black squares) for additional models with the lines of identity.

Changing the values of F-to-enter (F = 1.5) and F-to-remove (F = 1) resulted in an additional relation for *f_u_* and *f_b_*/*f_u_*. It was revealed that LUMO and IOPO are determinants for *f_u_*, and COAR and HOMO are determinants for *f_b_*/*f_u_*. These models are characterized by significant correlations, *R*^2^ = 0.68 and 0.74, and average predictive performances, *Q*^2^ = 0.32 and 0.52 for *f_u_* and *f_b_/f_u_,* respectively (Table 4). The relationships between predicted and observed values of *f_u_* and *f_b_*/*f_u_* are shown in Figure 3.

**Figure 4:**
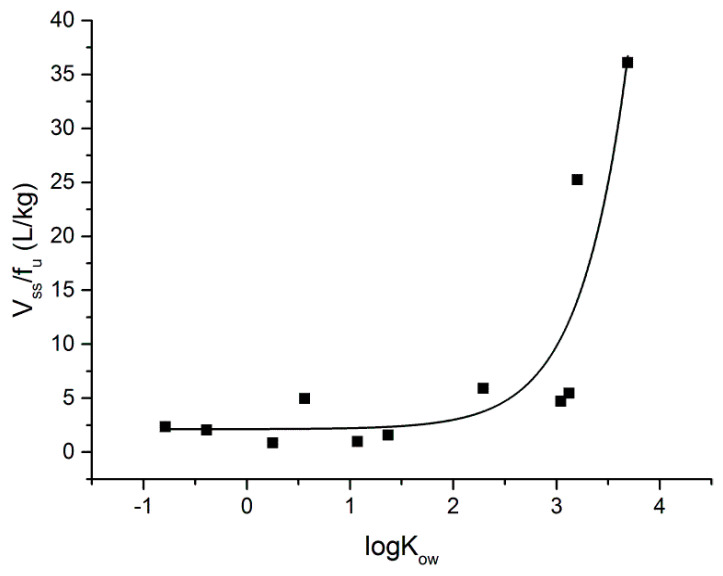
Experimental *V_ss_*/*f_u_* values vs. log*K_ow_* (black squares) with exponential fit (solid line).

Interestingly, the unbound volume of distribution *V_ss_*/*f_u_* vs. log*Kow* relation was well fitted (*R*^2^ = 0.83) by the exponential function (Figure 4).

**Figure 5:**
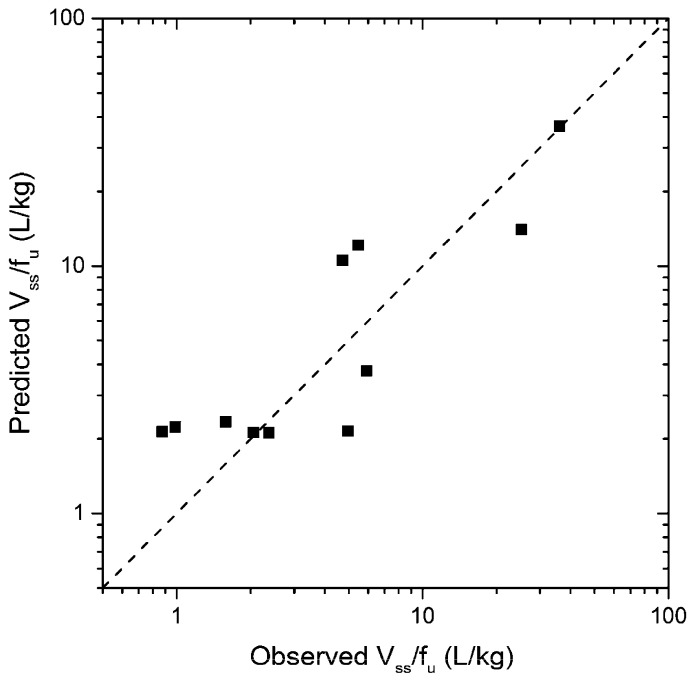
Predicted vs. actual (observed) *V_ss_*/*f_u_* values (black squares) with the line of identity.

LOOCV analysis showed a very good predictivity for this model (*Q*^2^ = 0.78, Table 3). Predicted vs. observed values of *V_ss_*/*f_u_* are shown in Figure 5.

## 4. Discussion

In the present study, the pharmacokinetic parameters of Theo and its derivatives (GR-1 to GR-8) as well as PTX and (R)-LSF were correlated with their physicochemical descriptors. Pharmacokinetic parameters were calculated based on the concentration vs. time profile data obtained in the experiments performed in rats. The results of the pharmacokinetic studies revealed that all studied compounds are characterized by different pharmacokinetic profiles. The estimated volume of distribution at the steady state (*V_ss_*) of the tested compounds varied from 0.33 to 2.21 L/kg. The lowest value of *V_ss_* was observed for the compound GR-8 (0.33 L/kg), which may indicate that its distribution is restricted solely to the body water. At the same time, a high value of *V_ss_* was observed for the compounds GR-4 > GR-1 > GR-3 > GR-2 (2.21–1.39 L/kg, Table 1). The presented above values of *V_ss_*, which significantly exceeded the volume of total rat blood, indicated that the investigated compounds (GR-1–GR-4) may easily penetrate through biological membranes and may be effectively distributed to many organs and tissues, including the central nervous system.

Taking into account the chemical structures of studied compounds, a strong positive influence of the substituent in the phenyl ring of the structure of compounds GR-2, GR-3, and GR-4 with the same five-carbon unit on the value of *V_ss_* may be observed. The highest *V_ss_* were estimated for compounds GR-1 and GR-4 with a 3-Cl substituent in a phenyl ring. Compound GR-3 with 2-OCH3 moiety displayed a lower *V_ss_* than its 3-Cl analog (GR-4) (Table 1). The unsubstituted in a phenyl ring compound GR-2 showed the lowest value of this parameter within the set of respective structural analogs. The elongation of the linker length between Theo core and phenylpiperazinyl fragment from four- to five-carbon slightly increased compound *V_ss_* (GR-1 vs. GR-4). The introduction of phenylpiperazinylalkyl substituent with lipophilic properties in a 7 position of Theo significantly increased *V_ss_* in comparison with parent Theo (Table 1). On the other hand, the 7,8-disubstituted Theo derivatives (GR-5–GR-8; series II) with peripheral analgesic activity displayed similar (GR-5 and GR-7) or lower (GR-6 and GR-8) volumes of distribution than those of reference xanthine (Theo) and PTX and (R)-LSF. The theophyllin-7-yl butanoic acid derivatives with hydrazide or benzylamide moieties, or free carboxylic groups (GR-5–GR-7) displayed lower *V_ss_* values (0.33–0.78 L/kg) in comparison with series I. Among the compounds of series II, GR-5 with a hydrazide moiety and GR-7 with a benzylamide group exerted the same *V_ss_*. In turn, GR-6 with a free carboxylic group displayed *V_ss_* lower by 25%. Compound GR-8 as a uric acid derivative showed the lowest *V_ss_* in this set of compounds.

Compounds GR-1, GR-4, GR-7, and GR-8 were highly bound to the rat plasma proteins, and the values of percent of plasma protein binding (86–94%) were comparable to this value for the well-known synthetic derivative of Theo–diprophyllinum (84–90%) [19,20]. Compounds GR-2 and GR-3 showed average binding to plasma proteins in the range of 71–72%, which is similar to that of Theo (65%). In contrast, the compound GR-5 was bound to plasma proteins in only 11%, which was close to the binding of (R)-LSF (21%). The rat plasma clearances for GR-1–GR-5 were similar and ranged from 23.6 to 33.1 mL/min/kg (Table 1). This parameter value was almost two times higher for GR-6, GR-7, and GR-8 (43.1–68.1 mL/min/kg) than for GR-1–GR-5. The lowest plasma clearance was observed for Theo (2.14 mL/min/kg). Based on the half-life value (*t*_0.5*λz*_), the tested compounds can be divided into two groups. The first group consisted of the compounds rapidly eliminated, such as GR-8 (*t*_0.5*λz*_ = 6.75 min), GR-7 (*t*_0.5*λz*_ = 15.2 min), GR-6 (*t*_0.5*λz*_ = 17.1 min), and PTX (*t*_0.5*λz*_ = 14.7 min). The second group of compounds, such as GR-1-GR-5, were eliminated at a much slower rate, with the terminal half-life in the range of 35.6 for GR-2 to 50 min for GR-4. In relation to the rat, the *t*_0.5*λz*_ is relatively long, and when extrapolated to humans, it may reach values in the order of hours. This, in turn, should allow administration of these compounds once or twice daily. The longest terminal half-life was observed for Theo (*t*_0.5*λz*_ = 236 min).

In the current study, QSPKR models were obtained for the prediction of five main pharmacokinetic parameters (*V_ss_*, *V_z_*, *λ_z_*, *CL*, and *f_u_*) of a series of eight Theo derivatives (GR-1–GR-8) and selected methylxanthines (PTX, (R)-LSF, and Theo). It should be noted that the present study was primarily focused on the development of methodology, and the number of compounds used for the modeling was limited. However, despite the relatively small dataset, the predictive ability of the QSPKR models as judged by leave-one-out cross-validation was fair (i.e., the difference between *R*^2^ and *Q*^2^ was 0.2 in almost every case). In the case of volume of distribution, the high value of the regression coefficient for log*Kow* is consistent with many previously published reports that high lipophilicity is associated with a large *V_ss_* value [21,22,23]. In the present study, log*Kow* was calculated using the KowWin program, where log*Kow* of a compound is estimated by summing all-atom/fragment contribution values and correction factors occurring in a chemical structure. In 1995, Meylan and Howard proved that the atom/fragment contribution method was superior to other comprehensive Log*P* estimation methods [24].

The volume of distribution at the steady state of a drug can be defined as:

(4) $$V_{ss}=V_{p}+\sum_{i=0}^{n}K_{pt,\;i}\cdot V_{t,\;i},$$

where *V_p_* is the plasma volume, and *K_pt,i_* and *V_t,i_* are the plasma tissue partition coefficient and the physiological volume of the *i*-th tissue. Since drug lipophilicity is a major determinant of *K_pt,i_* values, therefore, it has a great influence on the volume of distribution [25]. *V_ss_* can also be defined according to the Gillette equation [26]:

(5) $$V_{ss}=V_{B}+\left(\frac{f_{u}}{f_{uT}}\right)\cdot V_{T}$$

where *f_u_* and *f_uT_* are the free fraction of the drug in plasma and tissue, and *V_T_* is the sum of all tissue volumes. Thus, plasma protein and tissue binding are also important determinants of the *V_ss_* value. Traditionally, the *V_ss_* of free drug only has been linked with lipophilicity, particularly based on the role of this descriptor in tissue and protein binding. Modern QSPKR analysis frequently identifies lipophilicity as well as other steric and electronic molecular descriptors as important contributors to *V_ss_* [1]. In this study, log*D* emerged as an important contributor to the total *V_ss_*, *λ_z_*_,_ and *CL* of investigated compounds. The descriptor log*D*, encoding the lipophilicity of the ionizable compounds as it accounts for the pH dependence of a molecule in aqueous solution, appears to be the most significant determinant of *V_ss_*. There are four compounds with negative log*D* in the dataset. Two of them have small *V_ss_* (<0.5 L/kg). Oppositely, four of the five most lipophilic compounds (with log*D* > 2.3) have large *V_ss_* (>1.4 L/kg). The positive effect of log*D* on *V_ss_* has long been recognized [22,23,25,27]. This is not surprising, as good lipophilicity is required for many processes involved in drug distribution, such as membrane permeability, binding to tissue components, and accumulation in many organs and tissues. However, it can be seen that LUMO and HEFO are also important factors that are positively correlated with *λ_z_* and *CL* of studied compounds, whereas higher values of COAR are associated with larger *V_z_*. HEFO, a descriptor indicating the intrinsic energy of a compound, also had a high impact on the clearance of 1,4-dihydropyridine calcium channel blockers in humans [28] and adenosine A1 receptor agonists in rats [16].

It is believed that the unbound volume at steady-state is the most appropriate parameter to describe changes in lipophilicity and distribution within the homologous series of compounds [29]. The majority of studies reported a linear relationship between the logarithms of apparent volume of distribution and log*P* in homologous series of compounds [28,29,30,31,32]. However, the study covering a very large range of log*P* values using a homologous series of 5-n-alkyl-5 ethyl barbituric acids revealed a nonlinear behavior [33]. The results indicated that in a range of low log*P* values, log(*V_ss_*/*f_u_*) increased very slowly, whereas for high log*P* values, significant changes in log(*V_ss_*/*f_u_*) were observed. In the present study, Figure 4 illustrates a similar relationship between unbound volume (*V_ss_*/*f_u_*) and lipophilicity (log*Kow*) for a series of Theo derivatives (GR-1 to GR-8) and selected methylxanthines. The explanation of this phenomenon was proposed by Watanabe and Kozaki [22]. According to their theory, the drug distribution at pseudo-equilibrium may be modeled according to the following equation:

(6) $$V=V_{p}\left[1+k_{1}\left(\frac{V_{1}}{V_{p}}\right)+k_{2}\left(\frac{V_{2}}{V_{p}}\right)\right]$$

where *V_p_* is plasma space, *V*_1_ and *V*_2_ are lipid and nonlipid spaces, respectively, *k*_1_ and *k*_2_ are equilibrium constants relating the concentration in *V*_1_ and *V*_2_ to that in *V_p_*. After introducing the Collander-type relationship between *k* and *P* and grouping the constants, Equation (6) may be simplified to the following:

(7) $$logV=\log\left(a+bPc\right)$$

This equation indicates that the linear relationships between log*V* and log*P* observed in many studies are special cases of a more general Equation (7).

To our knowledge, this is the first study where QSPKR models were developed based on the in vivo data collected after the administration of xanthine derivatives. The results obtained using the leave-one-out cross-validation confirmed that the structure-pharmacokinetic relationships for this group of compounds were quite strong, and they can be useful for predicting the pharmacokinetic parameters of new xanthine-based compounds. However, the present study has several limitations. First, the number of molecules available for statistical analysis was rather limited; thus, the results may not be as robust as those obtained with a larger dataset and should be treated with caution. Nevertheless, the number of compounds analyzed in this study (11) is comparable with those in other QSPKR studies. For example, van der Graaf et al. [16] used 12 structurally related adenosine A_1_ receptor agonists for the analysis; Zhou et al. [28] developed QSPKR models for only 9 1,4-dihydropyridine calcium channel blockers, whereas Mager and Jusko [17] employed exactly the same number of corticosteroids for their evaluations as that in the present study. Another limitation of this work is the lack of external model validation. Due to the small number of compounds, the sample size was not large enough to derive an external group of compounds to further validate the model. The established relationships may require prospective validation in an independent group of xanthine derivatives in the same species. The new experiments may also be designed to revalidate the developed QSPKR models in other species commonly used in preclinical studies, such as, for example, mice or nonrodent species.

## 5. Conclusions

In conclusion, the statistical approaches to study QSPKR used in this investigation can capture the relationships between molecular descriptors and pharmacokinetic parameters of tested xanthines. Despite the relatively small dataset, the predictive performance of the developed QSPKR models was quite good. A larger dataset would be required to validate the current QSPKR models. This study’s results may help to guide the design and synthesis of new Theo derivatives with more favorable pharmacokinetic properties. The methodology employed in this study can also be used for other classes of compounds to develop robust QSPKR models in order to facilitate further integration of QSPKR in drug discovery and preclinical development. When coupled with pharmacodynamic models, it may be used for the prediction of the intensity and time course of the pharmacological responses of new compounds.

## Figures and Tables

**Table 1:** Pharmacokinetic parameters (as geometric means) of Theo, its derivatives (GR1 to GR-8), and other methylxanthines (PTX and (R)-LSF) (*n* = 4).

Compound	*V_z_* (L/kg)	*V_ss_* (L/kg)	*t*_0.5_*_λz_* (min)	*λ_z_* (1/min)	*CL* (mL/min/kg)	*f_b_*	*f_u_*	*MRT*_0–∞_ (min)
Theo	0.73	0.72	236	0.003	2.14	0.65	0.35	215
GR-1	2.17	2.05	45.4	0.015	33.1	0.92	0.08	62.1
GR-2	1.52	1.39	35.6	0.019	29.6	0.71	0.29	48.0
GR-3	1.70	1.55	49.8	0.014	23.6	0.72	0.28	62.1
GR-4	1.90	2.21	50.0	0.014	26.4	0.94	0.06	70.1
GR-5	1.43	0.78	39.0	0.018	25.3	0.11	0.89	29.2
GR-6	1.06	0.49	17.1	0.041	43.1	0.69	0.31	13.6
GR-7	0.95	0.78	15.2	0.046	43.2	0.87	0.13	19.0
GR-8	0.66	0.33	6.75	0.103	68.1	0.86	0.14	7.75
PTX	1.18	0.92	14.7	0.047	55.5	0.81	0.19	18.5
(R)-LSF	0.82	0.78	25.7	0.027	22.1	0.21	0.79	36.3

*V_z_*—volume of distribution based on the terminal phase, *V_ss_*—volume of distribution at steady-state, *t*_0.5/_*_λz_*—terminal half-life, *λ*_z—_first-order rate constant associated with the terminal (log-linear) portion of the curve, *CL*—total body clearance, *f_b_*—fraction bound to plasma proteins, *f_u_*—fraction unbound in plasma, *MRT*_0–∞_—mean residence time.

**Table 2:** Physicochemical descriptors of Theo, its derivatives (GR-1 to GR-8), and selected methylxanthines (PTX and (R)-LSF).

Compd	MW (g/mol)	HOMO (eV)	LUMO (eV)	HEFO (kcal)	IOPO (eV)	DIPO (Debye)	TOEN (eV)	CCR (keV)	NOFL	EE (keV)	log*Kow*	log*D*	COAR	COVO
Theo	180.2	−9.08	−0.38	4.44	9.08	3.27	29.63	10.18	34	−12.66	−0.39	−0.53	156.8	130.0
GR−1	430.9	−8.53	−0.34	35.39	8.53	2.95	49.30	35.21	80	−40.52	3.2	3.14	416.2	415.4
GR−2	410.5	−8.46	−0.29	39.12	8.46	3.80	45.06	34.81	80	−39.92	3.04	3.01	419.0	419.2
GR−3	440.5	−8.29	−0.29	3.30	8.29	3.78	55.65	39.23	86	−44.82	3.12	3.08	443.1	444.1
GR−4	445.0	−8.68	−0.30	32.73	8.68	3.42	44.45	36.53	83	−42.00	3.69	3.65	436.6	437.6
GR−5	324.3	−8.73	−0.20	−55.05	8.73	2.74	35.67	26.51	63	−30.97	0.25	−0.76	297.0	267.3
GR−6	296.3	−8.82	−0.26	−127.0	8.82	2.59	37.51	22.56	57	−26.75	1.37	−2.33	259.7	228.4
GR−7	385.4	−8.73	−0.18	−44.31	8.73	3.67	39.99	32.40	74	−37.47	2.29	2.29	351.4	315.6
GR−8	268.2	−9.05	−0.59	−120.3	9.05	2.09	21.64	19.76	51	−23.64	−0.79	−0.83	221.1	195.5
PTX	278.3	−9.02	−0.41	−48.20	9.02	4.59	34.55	20.19	54	−23.89	0.56	0.23	260.3	232.4
(R)-LSF	280.3	−8.87	−0.26	−63.42	8.87	2.44	37.52	20.73	55	−24.46	1.07	0.20	267.0	238.1

MW—molecular weight, HOMO—highest occupied molecular orbital, LUMO—lowest unoccupied molecular orbital, HEFO—heat of formation, IOPO—ionization potentials, DIPO—dipole moment, TOEN—total energy including electrostatics, CCR—core–core repulsion, NOFL—number of filled levels, EE—electronic energy, log*Kow*—log octanol-water partition coefficient, log*D*—log octanol–water partition coefficient corrected for the ionization state of the molecule at physiological pH, COAR—Connolly molecular surface area, COVO—Connolly surface volume.

**Table 3:** Summary of QSPKR models.

Pharmacokinetic Parameter	Equation	*Q* ^2^	*R* ^2^
*V_z_* (mL/kg)	*V_z_ =* 4.33 COAR *−* 104.47	0.597	0.717
*V_ss_* (mL/kg)	*V_ss_ =* 259.15 log*D* + 826.88	0.600	0.740
*λ_z_* (1/min)	*λ_z_ =* 0.00996 log*D −* 0.12584 LUMO *−* 0.00058 HEFO *−* 0.03690	0.710	0.908
*CL* (mL/min/kg)	*CL =* 8.20 log*D −* 65.58 LUMO *−* 0.39 HEFO *−* 7.53	0.335	0.737
*V_ss_*/*f_u_* (mL/kg)	*V_ss_*/*f_u_* = 2115.99 + 11.37 $e^{2.17\log K_{ow}}$	0.779	0.834

**Table 4:** Additional QSPKR models obtained after reducing the level of significance for descriptor entry and exit tests.

Pharmacokinetic Parameter	Equation	*Q* ^2^	*R* ^2^
*f_u_*	*f_u_* = −0.211 log*K_ow_* + 1.671 LUMO − 0.655 IOPO − 6.915	0.32	0.68
*f_b_*/*f_u_*	*f_b_*/*f_u_* = 0.098 COAR − 31.87 HOMO − 305.63	0.52	0.74

## Data Availability

The raw data supporting the conclusions of this article will be made available by the authors upon request.
